# Process Mining Methodology for Health Process Tracking Using Real-Time Indoor Location Systems

**DOI:** 10.3390/s151229769

**Published:** 2015-11-30

**Authors:** Carlos Fernandez-Llatas, Aroa Lizondo, Eduardo Monton, Jose-Miguel Benedi, Vicente Traver

**Affiliations:** 1 Instituto Universitario de Investigación de Aplicaciones de las Tecnologías de la Información y de las Comunicaciones Avanzadas (ITACA), Universitat Politecnica de Valencia, Camino de Vera S/N, Valencia 46022, Spain; aroalig@gmail.com (A.L.); vtraver@itaca.upv.es (V.T.); 2 Unidad Mixta de Reingeniería de Procesos Sociosanitarios (eRPSS), Instituto de Investigación Sanitaria del Hospital Universitario y Politecnico La Fe, Bulevar Sur S/N, Valencia 46026, Spain; 3 MySphera S.L. Ronda Auguste y Louis Lumiere 23, Nave 13, Parque Tecnologico, Paterna 46980, Spain; emonton@mysphera.com; 4 Pattern Recognition and Human Language Technology(PRHTL), Universitat Politecnica de Valencia, Camino de Vera S/N, Valencia 46022, Spain; jmbenedi@prhlt.upv.es

**Keywords:** process mining, indoor location systems, health process

## Abstract

The definition of efficient and accurate health processes in hospitals is crucial for ensuring an adequate quality of service. Knowing and improving the behavior of the surgical processes in a hospital can improve the number of patients that can be operated on using the same resources. However, the measure of this process is usually made in an obtrusive way, forcing nurses to get information and time data, affecting the proper process and generating inaccurate data due to human errors during the stressful journey of health staff in the operating theater. The use of indoor location systems can take time information about the process in an unobtrusive way, freeing nurses, allowing them to engage in purely welfare work. However, it is necessary to present these data in a understandable way for health professionals, who cannot deal with large amounts of historical localization log data. The use of process mining techniques can deal with this problem, offering an easily understandable view of the process. In this paper, we present a tool and a process mining-based methodology that, using indoor location systems, enables health staff not only to represent the process, but to know precise information about the deployment of the process in an unobtrusive and transparent way. We have successfully tested this tool in a real surgical area with 3613 patients during February, March and April of 2015.

## 1. Introduction

The application of business process standardization and analysis techniques has had a major impact on the global understanding about the concept of processes engineering [1]. Currently, quality management is associated with a complete standardization and continuous improvement within all the processes of organizations. Quality models, such as ISO9001 [2] or Capability Maturity Model Integration (CMMI) [3], have clearly identified the definition, monitoring and improvement of processes as the main issue to perform a correct quality plan. Although this idea was traditionally and mainly focused on enterprise environments, this idea has been exported to other areas. One of those is the case of health. Based on evidence-based medicine (EBM) principles [4,5], the scientific community is requesting the incorporation of clinical evidence to support the daily practice of health professionals. Nevertheless, the continuous evaluation of current evidence is unsustainable to be performed by individual professionals [6]. To deal with these challenges, EBM principles were traditionally implemented as clinical pathways [7]. Clinical pathways are protocols of clinical interventions, timely placed, written and agreed upon by a multidisciplinary team [8]. In this line, the main clinical pathway role is to limit healthcare variability in medicine, to improve clinician performance [9,10] and to avoid errors [11].

Despite the high quantity of clinical pathways available for health professionals in online digital libraries, like PUBMED or COCHRANE, its penetration into daily clinical routine is not as deep as expected [12]. This is because the clinical pathway deployment is a very complex problem to be solved [13], the difficulty of tracking the real execution of the formal protocols being one of the main barriers. The application of clinical pathways requires the continuous monitoring of the patient care cycle to not only evaluate the real deployment, but also to allow the discovery of inefficiencies or bottlenecks in the process, allowing their correction [14].

In this environment, information technologies (IT) are becoming the solution to allow the design and deployment of clinical pathways in a sustainable way [9]. For that, the deployment of clinical pathways through IT is currently taking more importance among the different health infrastructure decision makers. However, in several works [10,12,15], it is demonstrated that the use of IT in the deployment of clinical pathways improves the satisfaction of the health care staff, enhancing care process efficiency.

However, the deployment of clinical pathways can increase the bureaucratic workload, and problems can arise in relationships among general practitioners (GPs) and the local health authority [16]. In addition, the literature recommends that the measurement of the deployed processes should be considered as part of the clinical pathway [17]. However, this might increase the risk of rejection of the protocol. To avoid that, the selection of a non-intrusive method for gathering clinical pathway data and evaluating the correct execution of the process is vital to allow a sustainable deployment of the clinical pathways [16].

One of the methods that is currently used for unobtrusive monitoring is indoor location systems (ILS) (also known as real-time location systems (RTLS)). With the arrival of those infrastructures, a wide range of different applications of ILS solutions has appeared in the literature [18,19,20]. There are systems that use the localization to support the activity recognition of humans in smart homes [21], systems that monitor the daily activity of elderly in ambient assisted living (AAL) environments [22] and systems that create a movement behavior model of users in smart places [23]. In the case of clinical care, ILS systems have been used for unobtrusively measuring the time of treatment of hospitals for better management and improvement of the quality of service [24,25,26]. The idea of using ILS to gather process timing allows not only a precise and accurate, but also an unobtrusive and human error-safe way to measure the clinical pathways’ execution. Using these systems, it is possible to analyze the execution time of some critical processes [24] and to decide on further improvements.

However, performing simple statistics over the clinical pathway ILS might be not enough to achieve a global human understanding of the processes’ execution. New technologies are needed to take advantage of these data in order to offer a general view of the execution process, making it easier for humans to understand the process characteristics. This is the case of process mining. Process mining [27] is an emerging paradigm that uses the logs gathered from the execution of processes to infer human-readable models. Using this technology with clinical pathway ILS logs, it is possible to show a dynamic view of the behavior of the protocol. Our hypothesis is that this view can help in the real understanding of the process inefficiencies more easily than simple statistics.

In this paper, we propose a process mining-based methodology that, taking advantage of the clinical ILS data, infers a visual workflow to represent the real view of the clinical processes’ execution. This proposed system has been tested in the surgery area of the General Hospital of Valencia, analyzing the surgery processes in a real setting.

## 2. Related Work

Process mining [27] is a paradigm that comes from the business process management research field, which allows the discovery and graphical representation of human-understandable models that represent the real execution of a process. Process mining discovery algorithms use logs for creating workflows, which represent the processes’ possible paths and their associated statistics in a graphical way. The process mining methodology can offer an easy way for understanding the process, providing a view of the dynamical dimension of the execution of the process. The idea of using process mining for healthcare systems is relatively recent [28]. Traditionally, process mining covered three different ways to support the analysis of processes, discovery, conformance and enhancement [27].

Process discovery is a process mining technique that is based on analyzing existing logs in order to represent the model of the process. In this line, there are works intended to characterize patterns of clinical pathways [29], discover process patterns in different hospital departments or health centers [30] or analyzing aspects of specific illnesses [23,31], among others. On the other hand, process conformance aims at the analysis of process execution samples in order to determine if these conform to the model defined in the process. Unlike process discovery techniques, process conformance assumes that there is an *a priori* model and provide algorithms to assess the conformity of the process instances to the general model. In this way, there are works that use these techniques for outlier detection [32] or for assessing adherence to medical treatments [33], among others. Finally, process enhancement (also known as extension) is aimed at enriching the models with information collected from the execution samples. Like process conformance, this technique requires the existence of a previously-defined process model. An example of use is the enrichment of the process model with information about performance or time of use, highlighting specifically the most important patterns in the flow [23].

In order to use process mining for assessing professionals in daily practice, there are different academic (ProM [34]) and commercial applications (DISCO [35]) that can be used in several research fields. Both are powerful desktop-based tools that can be used for applying process mining techniques using a standard event log to perform its own research. ProM is the most used academically and a freely downloadable process mining tool. This tool not only contains implementations of the most classical process mining algorithms, but also allows the creation of plugins for adding new ones inside the tool. ProM has a set of process mining algorithms available that come with official tools, like the *α*-algorithm [36], genetic process mining [37] or Heuristic Miner [38], or, as a plugin, like the parallel activity log inference algorithm (PALIA) [39].

However, web-based hospital information systems are usually preferred instead to desktop-based systems [40,41]. IT departments sometimes discourage the use of desktop-based tools, not only for maintenance, but also for security reasons. In fact, in some hospitals, the installation of non-corporate software is prohibited in order to avoid problems with viruses or hacker attacks. In this way, the integration of process mining desktop-based kinds of tools in the daily practice of healthcare professionals has a risk of breakdown due to the impossibility of using this tool in corporate computers and the necessity to start a formal query and anonymization protocol to legally transfer the data to another computer to get access to each one of the log files. For that, in order to allow the integration of the tool within hospital information systems, the use of web-based tools is highly recommended.

Process mining has problems with unstructured models, like healthcare traditional systems [28]. One of the most important problems dealing with the application of process mining tools is known as the spaghetti effect problem [27]. The spaghetti effect problem creates large and difficult to understand processes due to the inherent characteristics of the problem to be solved. In healthcare, the high variability of the medical processes, as well as the noise and the difficulties in accessing homogeneous health data make their usability very poor [42].

In this way, in order to take advantage of process mining techniques, it is necessary not only to provide methodologies and algorithms to increase the usability and understandability of health professionals, but also to find clean suitable data for analyzing the healthcare processes [43].

## 3. Materials and Methods

On some occasions, especially in health, the deployed process could differ significantly from the designed or even the perceived process. The causes of this are several: untrained professionals, lack of protocols, lack of real communication between managers and health experts, lack of infrastructure, *etc*. In order to solve this, it is necessary to provide methods that enable health professionals to measure processes in order to acquire knowledge of the process in which they are involved themselves, not only for correcting those discrepancies, but also to allow continuous improving of the process, adapting it to the real deployment context.

Measuring health processes has a large amount of advantages [17], not only in the knowledge of the process itself, but also in the improvement of the quality of service, increasing efficiency, improving treatments, avoiding errors, *etc*. In previous works, we presented an interactive pattern recognition methodology oriented toward health [14]. Interactive pattern recognition is an iterative methodology that uses pattern recognition algorithms in combination with experts corrections in order to provide more easily deployable models. This new methodology requires that pattern recognition algorithms are understood by humans. Process mining techniques are pattern recognition tools specifically designed to be used by human experts. The use of process mining techniques can support health professionals in discovery, the analysis of conformance and provide an enhancement of the process visualization in each iteration. For this, in this paper, we present the use of process mining as an adequate pattern recognition technique for this challenge.

Enabling the deployment of continuous process improvement in a certain scenario requires collecting punctual, reliable and significant data for a continuous process monitoring. In addition, these data should be recovered in as an unobtrusive way as possible, in order to avoid the appraisal of the process affecting the process itself. However, obtaining quality data from hospital databases is not always possible. The data are obtained from different data sources created by different health professionals and exposed to bias and subjective contexts. Due to this, there may exist an undesirable variability that can result in the increase of the spaghetti effect [42]. This effect can make the understanding of the processes provided by process mining techniques more difficult, making the system unusable. In this way, the use of homogeneous and objective data sources can make the understanding of the hospital processes easier.

Some hospitals and health centers have installed indoor location systems in order to increase the security of their patients and medical tools. This is the case of General Hospital of Valencia (Spain), which has developed an innovative punctual information system for relatives of surgical patients in collaboration with the enterprise MySphera. This system informs in real time about the status of the patient that is in a surgical process, allowing relatives to know the moment when the family member enters or leaves the surgical room. Although this system itself provides a direct added value to relatives of surgical patients while they are in the waiting room, this information also can be used to get more information about the surgical process.

However, the large quantity of data generated by the system makes it complex for health managers to take advantage of these data. In order to be effective in the evaluation process, it is necessary to create condensed and easy to understand views to help professionals in the understanding of the deployment problems of their processes. However, process mining techniques, can provide an understandable view of the process deployed. In this paper, we propose an interactive pattern recognition methodology based on the application of process mining techniques using ILS data.

The selection of an easy to use and accessible tool for professionals that could be integrated within their daily process is key for its success. The technology used should not be a barrier for the adequate deployment of the system. In fact, improving the understandability and usability of process mining techniques is an open challenge [43]. For those reasons, we have developed a tool specifically designed to use process mining techniques with ILS data in hospital environments in order to facilitate its use for doctors, avoiding the barriers for usability of existing tools by inexperienced users, as well as the installation and maintenance barriers of IT departments in health centers.

For the selection of the process discovery algorithm, we have analyzed mainly two algorithms: the heuristic miner [38] and PALIA [39]. These algorithms used finite state models for process representation. In the healthcare case, finite state models are used because they are better understood by health professionals [39].

The heuristic miner algorithm [38] is simple and quick and generates a dependency graph from the events occurring in a workflow log. This algorithm not only discovers the flow followed by the instances, but also is able to provide the dependency probability between events that is usually specified graphically in the arrows between two nodes. The heuristic miner algorithm is specifically for event-based data and assumes that there are no parallel nodes.

PALIA [39] uses a compendium of syntactical pattern recognition techniques to construct a formal automaton, called timed parallel automaton (TPA) [44], that is expressively equivalent to a safe Petri net for representing the process. PALIA can deal with parallel actions because it performs an analysis of the time in the creation of the dependencies among nodes. In addition, although PALIA can deal successfully with event-based data [23,39], PALIA is specifically designed to deal with activities. An activity provides, in addition to the start and end event of the workflow log, the result of the activity. This is important in health processes, because it provides a condensed view of the decision made in health protocols. For example, we suppose a medical protocol that explains the use of a drug (aspirin) when a patient has a fever. Using only event data, it is possible to infer that, after taking their temperature, some patients take a pill and others do not. However, using the result of the event of taking the temperature (fever or not fever), PALIA can annotate arrows between nodes. This allows PALIA to define a dependency among actions depending on their results, which is not available in other algorithms.

For our current problem, both algorithms can deal perfectly with ILS. There is no possibility of parallel actions, and the entered data are event based. For that, we believe that the results achieved using both algorithms would be similar. However, we have selected the PALIA process mining algorithm because, according to our past experiments, it has a generally better behavior [23,39]. In addition, the use of PALIA allow us to be more open in the case of the enrichment of the entry data in the future, mixing the current corpus with specific hospital data.

## 4. MySphera ILS System

The General Hospital of Valencia has installed in its surgery area an indoor location system (ILS) provided by MySphera. MySphera offers an active RFID-based system composed of a server and beacons that are installed in the areas where the user should be located. When a patient enters the surgery process, an identification bracelet with an active tag is placed on his/her wrist. Each bracelet has a unique number, which identifies the user. The bracelets connect periodically to the beacons through the ZigBee protocol, sending the bracelet identifier and an estimation of the distance of the beacon to the bracelet in a periodic and continuous way.

**Figure 1:**
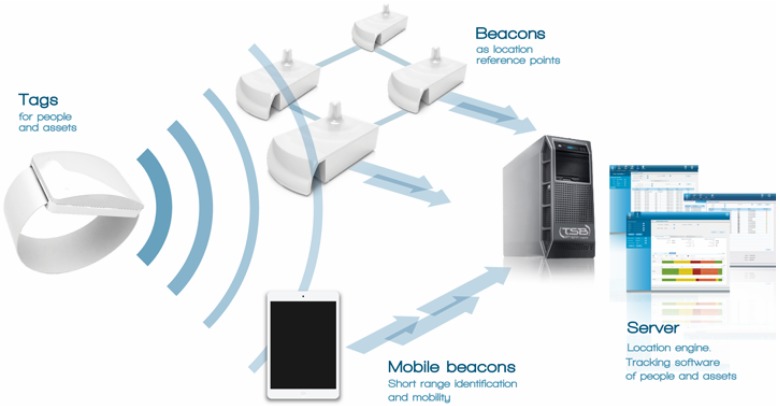
Architecture of the MySphera system [45]. Reproduced with permission from MySphera${}^{®}.$

The MySphera ILS architecture is shown in Figure 1. This system has the capability of providing a log of the localization changes for each one of the installed beacons in the surgery area. The different parts of the MySphera system are specified as follows:

- Tags are devices that allow locating the persons and objects that wear it. Tags periodically send frames (in a configurable period) based on the ZigBee standard technology. Frames are received by the beacons. The modulation used by ZigBee for transmitting data makes a low sensibility in reception possible, which means that the transmission power can be very low. In addition, the specifications of the protocol define very short frames, allowing the device to stay in a very low power consumption mode most of the time. Therefore, the tag has a very low power consumption, making tags that are smaller and having greater autonomy possible. On another hand, the use of a standard technology reduces the cost of the active asset. Tags are reusable, waterproof IP67(water resistant to 1 m), shock resistant to a three-meter fall and have a battery life (configurable depending on the periodicity of the location) between two and three years.
- Beacons receive the frames sent by tags, calculate the received signal strength indication (RSSI) and forward the data to the SPHERAONE location server. The installation of the beacons is carried out in fixed points along the areas where location coverage is needed, usually using supports fixed to the ceiling, and connected to the server using Ethernet cable (Cat. 6). Switches and the rest of the electronics network should provide power over Ethernet to supply power to beacons.
- Mobile beacons are USB adapters that allow the identification of tags using a portable device (for example, a tablet). This function is independent of the beacon location infrastructure. Tag desktop readers are also available, which are USB-based readers to be used on desktops, using USB to connect to a PC. This tag reader allows the reading of tags for its assignation to a person or an asset. The assignation process is to link the tag read by the desktop reader to the identifier of the asset or person. This assignation can be carried out using MYHOSPITAL software or integrated in the hospital information system (HIS) of the hospital.
- The MySphera server has two main software modules installed.
  On the one hand, SPHERAONE is the name of the location server. It includes the location algorithm that calculates the position of the tag with respect to predefined location areas, using the data received from the beacons. The Admin Center java application is the interface for the configuration and management of SPHERAONE server. It allows one to manage server parameters and to monitor the operation of the different devices of the RTLS system (battery, state, *etc*.);
  On the other hand, MYHOSPITAL is specialized software for the identification, location and tracking of patients, staff and assets in hospital environments. The background of MYSPHERA in the health sector is reflected in the development of software that provides much more than the location of objects and persons, offering a tool for the improvement of processes and a more efficient management of the hospital resources.

## 5. PALIA ILS Suite Web Tool

In this paper, we present a web process mining tool specifically designed for dealing with ILS data in healthcare environments. This tool provides discovery, conformance analysis and enhancement of the graphical view of the whole process through the use of process mining algorithms over localization events.

**Figure 2:**
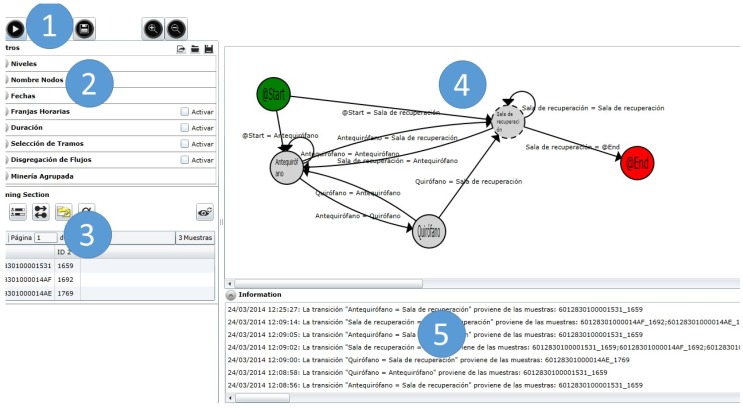
Screenshot of the parallel activity log inference algorithm (PALIA) indoor location system (ILS) suite tool.

Figure 2 shows a screenshot of the PALIA ILS suite, which is a web-based application developed in C# over Silverlight that allows users not only to execute process mining experiments using PALIA, but also to apply filters over the data, as well as rendering maps over inferred workflows.

The PALIA ILS suite user interface is divided into five areas:

- Area 1 is the main menu; in this area, the user can interact with the application executing new experiments, saving the obtained workflows for further analysis.
- Area 2 is the filtering area. This filtering area selects the data that will be used for process discovery, which will infer the flow representing the corpus using PALIA. In this area, it is possible to filter the data by type of patient, group nodes, specific dates, *etc*. Before an experiment starts, filters are configured to select the data that will be used for process mining. In this area, we have implemented specifically-designed filters for ILS systems. There are filters for grouping and renaming areas, for selecting dates, for selecting nodes with a specific maximum or minimum duration and also for selecting specific sections between two nodes inside the ILS flow.
- Area 3 hosts the mining render area. In this area, some conformance and enhancement algorithms have been implemented to be applied after the process discovery. These algorithms produce a rendering of the workflow inferred to provide an improved view of the discovered process. In this area, we have implemented general algorithms configured to be used in the ILS problem. We have conformance algorithms for comparing flows followed in the past with current ones or comparing ILS samples with discovered flows. Furthermore, we have enhancement algorithms for detecting jumps that allow technicians to correct undesired ILS errors; heat maps representing the most common paths and the percentage of the duration of each stage according to color codes; and very specific algorithms, like occupation maps, that allow one to show the current occupation of a room over time. Furthermore, in this area, the user can configure the visibility of tool tips that offer specific data of each node (number of occurrences, duration by occurrence, percentage of occurrences, *etc*.) or transition (percentage of occurrence by start node, number of occurrences, *etc*.).
- Area 4 is the workflow visualization window. Through this window, the user not only can accommodate the visualization by moving the nodes using drag and drop procedures, but also can obtain more statistical information of all of the structures, placing the cursor over arrows or nodes, or even mixing dynamical and statistical information fixing the tool tips.
- Area 5 is a text log with the output of the algorithms. Mining status, errors and warnings are displayed to the user in this window. This area can be collapsed in case more space is needed for the workflow visualization window.

## 6. Using Process Mining Techniques with ILS Data

The main objective of this paper is the analysis and evaluation of the combination of localization events with process mining techniques for allowing the deployment of an interactive pattern recognition methodology over health processes. In order to do so, an analysis flow over the ILS data gathered from the MySphera system and processed by the PALIA ILS suite was defined.

**Figure 3:**
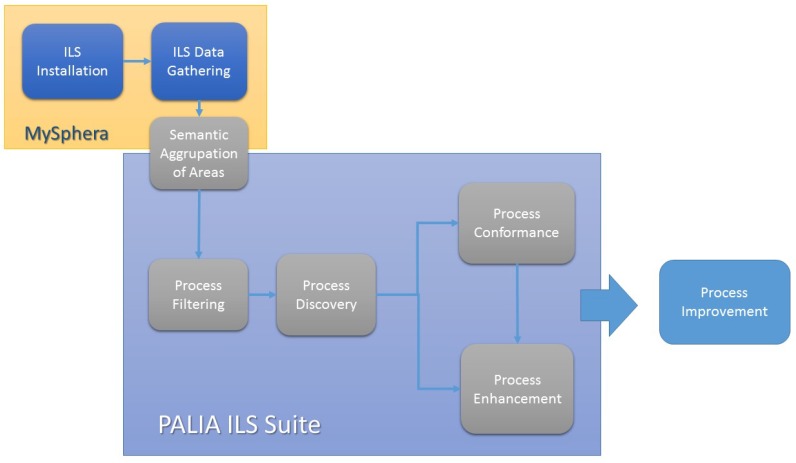
Scheme of the methodology.

Figure 3 shows the defined flow and the applications that take care of this in the deployed system.

The MySphera system covers the first stages of the analysis process. The first stage is the installation of the ILS system. In this case, MySphera technicians have installed beacons in all areas of the surgical department of the General Hospital of Valencia. Those beacons detect the presence of tags that are associated with each of the patients that are admitted. Each position of each patient in each moment in time is collected and stored in a central server. Once enough data have been collected, the next step is to group the beacons semantically depending on their represented area. This group is made in two dimensions, physical and logical. MySphera provides a categorization of the beacons depending on their positioning (for example, if two beacons are in the same room). In this way, MySphera provides a hierarchical categorization of all of the beacons. However, a more fine-grained categorization might be performed depending on the process discovery desired. For example, we can group specific operating rooms that are used for specific surgeries in one node in order to have a high level idea of the use of these rooms in the surgical process. This more specific grouping is made by health professionals in the PALIA ILS suite.

Using the data available, the PALIA ILS suite provides filtering options and algorithms for process discovery, conformance and enhancement. The first step that health professionals follow using the PALIA ILS suite is to select the samples, and sections of samples, that will be used to perform a process discovery. In order to do that, the users simple select and configure the filters of the filtering area of the tool. This is the process filtering stage. Then, PALIA is executed in the process discovery stage, producing a flow representing the process followed by the patients according to the restrictions configured in the process filtering step. Once the workflow has been inferred, the next step is to show enhanced information of the process and/or compare the current process with others. These stages of the methodology are performed with the algorithms available in the mining render area of the PALIA ILS suite. Health professionals can save and compare the workflows of previously-performed inferences, as well as to get heat maps with duration and occupation information.

Using this in an iterative way, it is possible not only to have a better view of the health process behavior that can be used for process improvement, but also to detect inconsistencies and failures in the ILS. That allows one to detect the more problematic areas and enables ILS technicians to improve the system effectiveness in these areas.

## 7. Characterizing the Surgical Process at General Hospital of Valencia

The clinical surgery process defines the protocol followed by a patient in his/her operation. In this section, the surgical stages and type of patients are described. Therefore, attending to the type of patient, it is possible to divide the surgical processes into four:

- Post-hospitalization surgery (PS): These patients are not hospitalized before the surgery, but the complexity of the operation requires a post-surgery hospitalization.
- Outpatient surgery (OS): Patients that have simple operations, not requiring hospitalization before or after the surgical process.
- Hospitalized patient surgery (HS): Patients that are having an operation while being hospitalized.
- Non-programmed patient surgery (NS): These patients do not have programmed surgeries, but due to unusual situations (like vital emergencies, foreign patients that are not in the HIS, *etc*.), have not been previously codified in the first stage.

**Figure 4:**
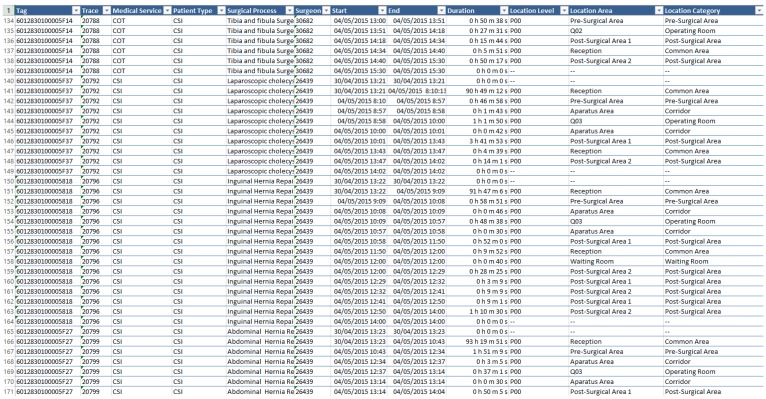
Example of data gathered from the ILS system.

MySphera produces CSV files that have in each line a positioning value for each tag. Figure 4 shows an example of the localization events provided by MySphera. In each line, the following data are available:

- Tag identification: This is a hexadecimal unique number that identifies the tag that was located by the beacon.
- Patient identification: This is a number that represents the patient associated with the tag at the moment of localization.
- Start event time: This is a date and time value that represents the moment at which the patient reaches this localization.
- End event time: This is a date and time value that represents the moment at which the patient disappeared from this localization.
- Location area categories: This is a set of texts that represents the localization area. There are three texts representing the location area categories: the location level that represents the building and floor, the area category that represents the kind of area, for example operating room, and the location area that defines a specific room, for example Operating Room 5.
- Sample metadata: In order to allow the performing of specific filtering, some metadata are added. For our problem, MySphera offers four specific fields. The origin of medical service allows filtering the samples by the medical services that were ordered for the intervention; the type of patient defines the type of surgical process (PS, OS, HS or NS); the surgical procedure defines the performed intervention; the surgeon ID is a number identifying the surgeon that performs the intervention.

The PALIA ILS suite requires a CSV file with a header that specifies the content of each one of the columns using specific keywords: ID for patient identification; START for the start event date and time information; END for the end event date and time information; EXCLUDE to ignore a column; ACTIVITY for location area categories; and SAMPLE for metadata information. In this way, the PALIA ILS suite can filter by future modifications of the entry corpus that are added to the metadata.

Through MySphera localization logs, as we can infer the actions happening at each one of the areas, it is easy to group the beacons depending on the stages of the process. Therefore, according to the steps of the process followed by each patient, is it possible to group the localization events of the surgical area into process stages. For our experiment, we have grouped the location areas in the following stages:

- Preparing: The preparing stage refers to the moment when the patient is being prepared for surgery. This refers to the location events occurring in the special room dedicated to this function.
- Surgery: This is the core stage of the process, during the precise intervention time in the operating room. This stage collects the location events occurred in the set of operating rooms available at the hospital.
- Recovery: In this stage, the patient is under surveillance just after the surgery.
- ICU: If the patient suffers a complication or in especially complex surgery cases, the patient is moved to the intensive care unit (ICU) to provide him/her special intensive care.
- Locker room: For those cases that do not require patient hospitalization after the surgery, the patient has a special area for dressing himself/herself with privacy.
- Adapting: This stage is a specific recovery stage for outpatient surgeries where medical staff makes a special follow-up of the intervention before the patient’s discharge, enabling him/her to go home.

In this paper, PALIA will be used in order to show the dynamic behavior of the care processes depending on the type of patients. To do that, the localization stages will be analyzed in order to generate a visual workflow that shows very easily the flow followed by patients, highlighting the most common paths. This is achieved using heat maps to support the understandability of the process.

## 8. Experimental Results

In order to test our system, we have validated our application in a real scenario. We have used the patients’ localization’s data available from the surgery area of the General Hospital of Valencia (Spain). Data were collected during February, March and April 2015. Table 1 shows numerically the size of the corpus used. During the three months of the pilot, we have collected the trace of 3613 surgical patients. That means a total of 39,364 localization events that have been grouped into 16,009 localization area changes. Our hypothesis is that the use of process mining algorithms allows one to characterize the dynamic behavior in a human-understandable view. Performing a detailed review of how the surgery protocol is happening at the hospital is out of the scope of this paper. The main objective of this paper is to demonstrate the potential of the combination of ILS systems with process mining techniques.

**Table 1:** Statistics of the corpus used for the experiment.

		February	March	April	Total
Total Patients		1263	1124	1226	3613
Samples by Patient Type	PS	308	303	378	989
	OS	827	712	696	2235
	HS	126	106	128	360
	NS	2	3	24	29
Number of States		5776	49,454	5288	16,009
Localization Events		14,244	12,564	12,556	39,364

**Figure 5:**
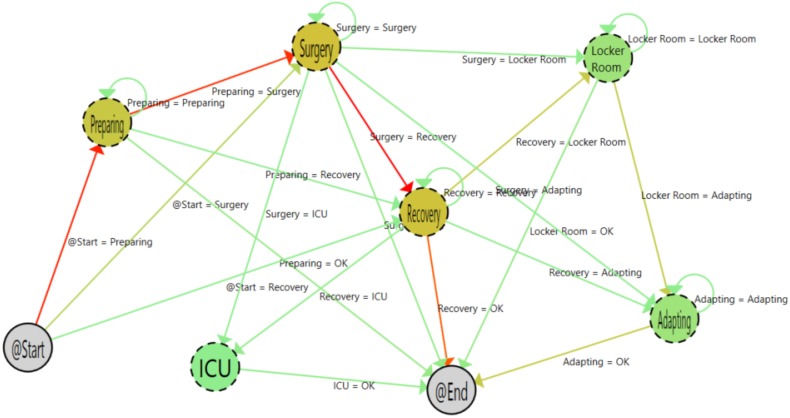
Process with all samples.

Figure 5 shows the result of the algorithms with the 3613 samples. As is shown, it is easy to understand the flow followed by patients. Furthermore, thanks to the color codes of the heat map algorithm, the most common path followed by patients is highlighted. The color gradient changes from green to red. On the one hand, for the transitions, the redder the color, the greaterthe number of times that this transition occurs. On the other hand, for states, the redder the color, the greater the time spent by the user in the state.

**Figure 6:**
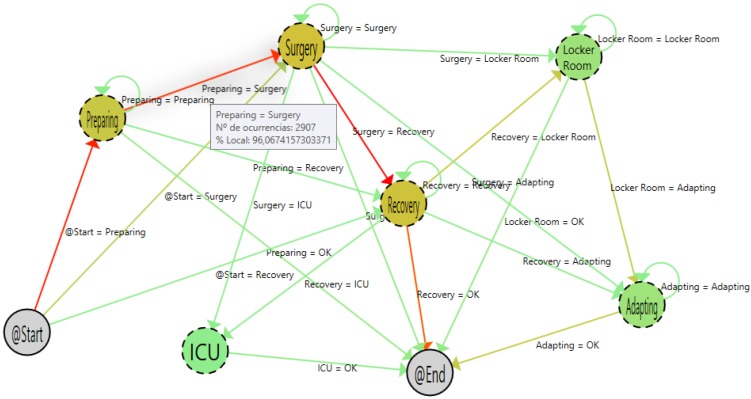
Detail of the tool tip.

Moving the cursor over the arrows, it is possible to have specific information about the transitions among states. Figure 6 shows the detailed view of the transitions among preparing and surgery states. As shown, during the pilot, 2907 patients (96.07% of the total amount) that were in the preparing state went to surgery. In the same way, if the user moves the cursor to other arrows that begin in the preparing state, the information that the PALIA suite offers is that 113 patients (3.73%) left the surgical area, and two patients (0.06%) went directly to recovery.

**Figure 7:**
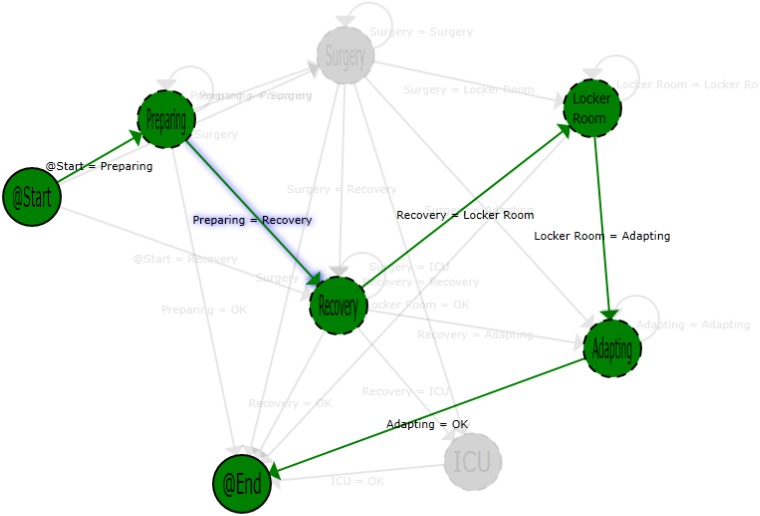
Path view of a single patient process.

The patients that left the area were those that had a programmed intervention that was canceled for some reasons. The shift from preparing to recovery is a more strange case. Using the application, it is possible to filter such specific cases and analyze what happened, following the whole patient’s path. Figure 7 shows a single path of a patient that makes a transition from the preparing to the recovery area. Using that information in combination with the hospital information system (HIS), this case can be analyzed in order to detect protocol failures or inefficiencies. In this specific case, the shift can be explained due to the patient suffering a sudden complication in the preparation phase, the intervention being canceled and the patient staying in the recovery area until he or she felt better. When that happens, he or she has left the surgical area.

**Figure 8:**
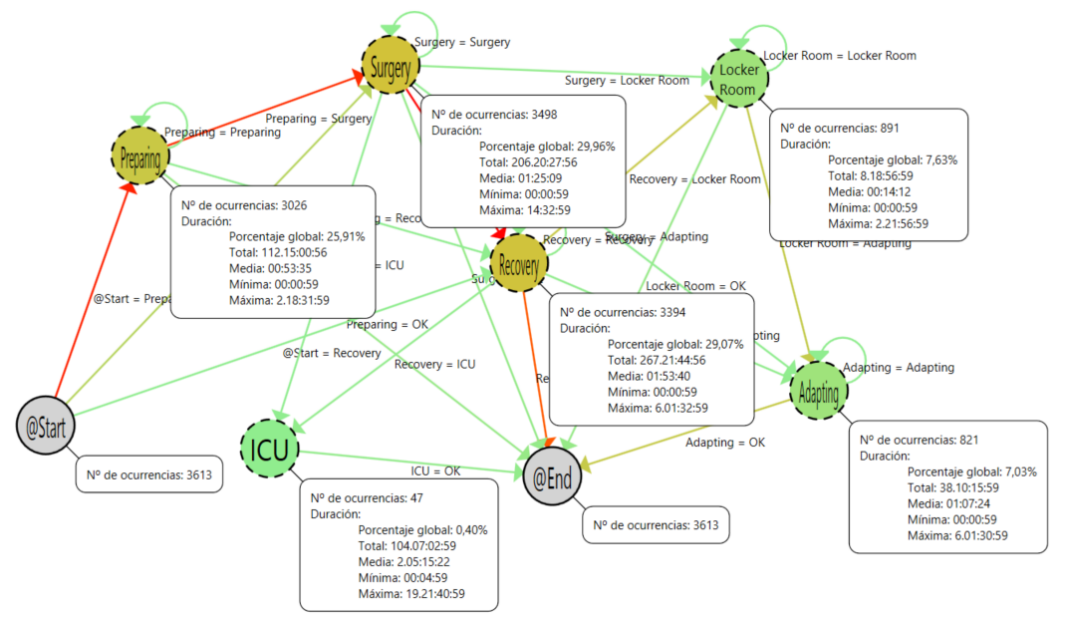
Process with all samples and fixed tool tips.

In the mining render area, there is a command to fix the nodes and the tool tips in order to allow a quick visual comparison among different stages. In Figure 8, a screenshot of this functionality is presented. In addition to the number of occurrences, the tool tip of the localization nodes includes information about the duration of the stay of the patients in each step of the process. A summary of this information is presented in Table 2. With this information, it is possible to analyze the timing of the process in order to detect inefficiencies and bottlenecks.

**Table 2:** All patients.

	*N*	Average Time	Time (%)
Preparing	3026	0:53:35	15.2
Surgery	3498	1:25:09	27.9
Recovery	3394	1:53:04	36.3
ICU	47	2 d 05:12:22	14.1
Locker Room	891	0:14:12	1.2
Adapting	821	1:07:24	5.2

Using the filtering area, it is possible to select the focus of the process to be shown. The filtering process selects only the samples of patients with such specific characteristics. In our experiment, we have filtered by type of patient in order to show how the application detects the specific issues of each kind of process.

**Figure 9:**
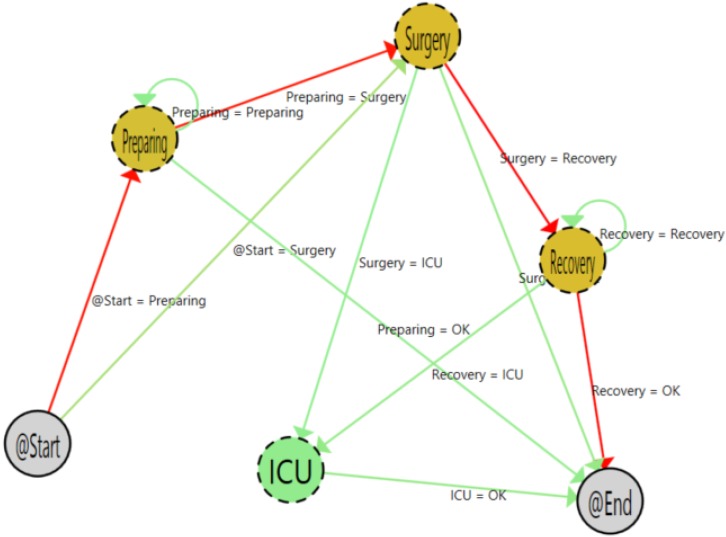
Post-hospitalized patient process.

Figure 9 shows the workflow as a result of filtering by post-hospitalized patient type. At first sight, it is clear that most of the time, the flow follows the normal path (preparing, surgery, recovery). It is also easy to see the great difference among the normal flow and the patients with medical complications who stay in the intensive care unit after the surgery. Additionally, it is easy to understand in the flow inferred that there is no presence in the locker room and adapting areas. This is normal, because hospitalized patients go directly to their hospital room after the surgery.

Analyzing the area states (Table 3), it is easy to show that this kind of patient suffers more complex interventions, and that is because he or she spends more time in the surgery and recovery areas than the global average (compared to Table 2), keeping a similar preparing time.

**Table 3:** Post-hospitalized patients states.

	*N*	Average Time	Time (%)
Preparing	893	0:57:39	10
Surgery	977	2:18:59	26.4
Recovery	956	3:28:16	38.7
ICU	29	3 d 1:39:51	24.9
Locker Room	-	-	-
Adapting	-	-	-

**Figure 10:**
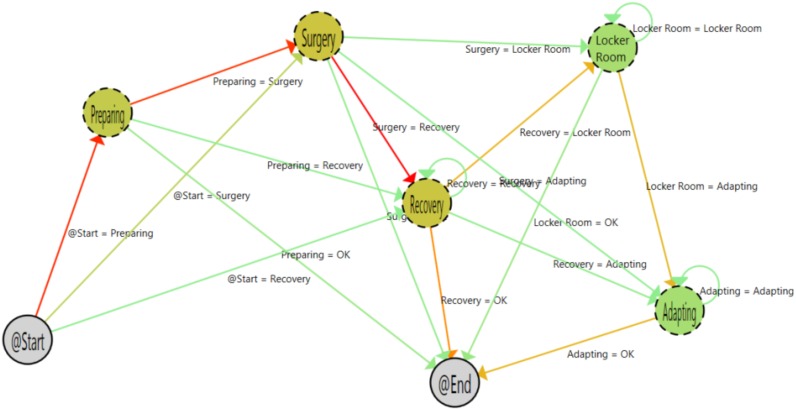
Outpatient process samples.

Figure 10 shows the inferred process of outpatient interventions. For this kind of operation, the adapting and locker room steps appear on the path. This is correct, as these patients are discharged on the same day when there is no problem after the intervention. The ICU phase was not needed for any outpatient surgery, as during the pilot, there were no complications for outpatient surgeries.

Table 4 shows the states associated with the outpatient interventions. As expected, the surgery and recovery phases take less time than for post-hospitalization patients. This is because outpatient surgeries are clearly less complex and less time consuming.

**Table 4:** Outpatients states.

	*N*	Average Time	Time (%)
Preparing	1828	0:49:38	23.9
Surgery	2155	0:46:52	26.6
Recovery	2095	0:57:00	31.5
ICU	-	-	-
Locker Room	891	0:14:12	3.3
Adapting	821	1:07:24	14.6

**Figure 11:**
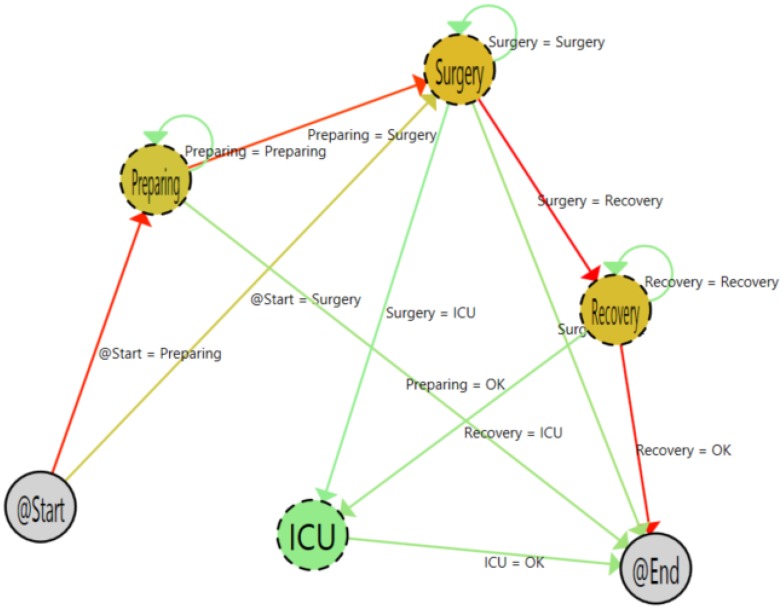
Hospitalized patient process.

Figure 9 shows the inferred graph for hospitalized patients. As shown, the graph is very similar to post-hospitalization patients (Figure 11). However, there is a subtle difference in the starting arrows. The transition between the start and surgery phase is darker for the hospitalized patients (23.33%) than in the post-hospitalized patients (9.8%). That shows that patients that are already in the hospital can be better scheduled for entry in the surgery room.

According to the stay of the patients in each location, Table 5 shows that, as expected, the consumed time for hospitalized patients in the surgery and recovery phases is very similar to the post-hospitalized patient case.

**Table 5:** Hospitalized patients.

	*N*	Average Time	Time (%)
Preparing	279	1:08:19	11.6%
Surgery	337	2:56:30	36.1%
Recovery	314	3:24:08	38.9%
ICU	18	20:22:36	13.4%
Locker Room	-	-	-
Adapting	-	-	-

**Figure 12:**
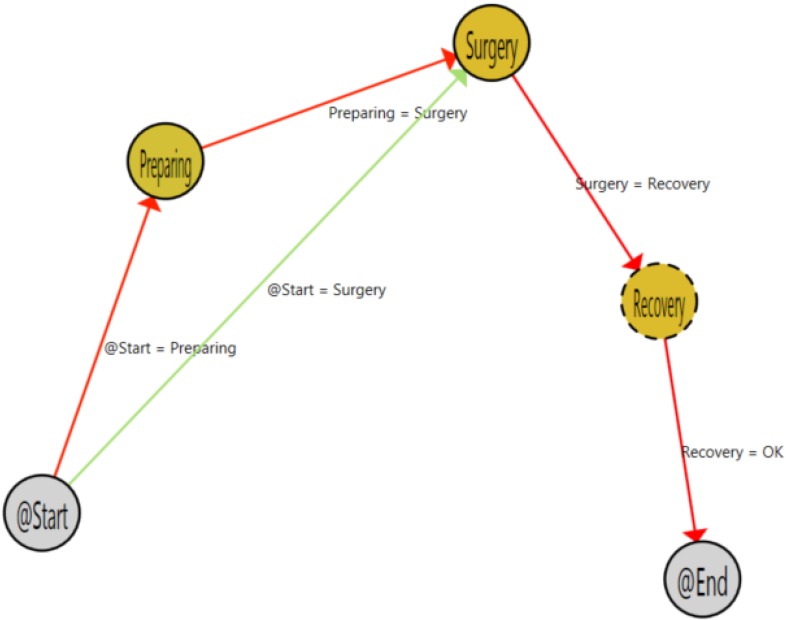
Process with other samples.

Figure 12 and Table 6 present the process and the states of patients that have not been previously assigned to any program.

**Table 6:** Other patients.

	*N*	Average Time	Time (%)
Preparing	26	0:33:23	15.5
Surgery	29	0:54:51	28.4
Recovery	29	1:48:35	56.1
ICU	-	-	-
Locker Room	-	-	-
Adapting	-	-	-

In order to evaluate the impact and usefulness of the application, six members of the health staff of the General Hospital of Valencia were asked to fill out a questionnaire. The questionnaire contained seven statements that health staff had to express their opinion about using the Likert scale (from one (strongly disagree) to five (strongly agree)). Table 7 shows the result of the questionnaire. As can be seen in the results, health professionals clearly agree on the utility of the system for their daily practice and consider it very easy to integrate within their daily workflow. They consider that the statistics presented are very interesting for decision support in the management of the surgical area. Although they agree about the understandability of the flow inferred, they asked for a view of the workflow over a 2D map of the surgery area in order to have a closer to reality view of the process.

**Table 7:** Results of the evaluation questionnaire for health staff (Likert scale).

Question	Value
I consider the application useful for my daily practice	4.6
The application can be integrated with my daily practice	4.4
The flow is easily understandable	3.4
I consider the application useful for surgical management	4.6
The visualization is clear	3.4
I prefer the flow inside a hospital map	4
I consider the presented statistics interesting	4.4

## 9. Conclusions and Discussion

According to our work, the application of process mining techniques in combination with ILS systems provides an easy to use and unobtrusive way to achieve a view of the deployed process. In our experiments, we have stated that the algorithm perfectly captures the features of the processes, showing them in an easy and understandable view that is accepted by the medical staff in a real environment. With this information, the health professionals and managers can achieve a real view of the problems that are currently happening in the analyzed area. This enables them the improvement of protocols with a better knowledge of the problems, increasing their efficiency and the probability of success for their further deployment in the real context.

The presented application allows one to go beyond the fact of inferring the general characteristics of the process. Using this application, it is possible to detect specific rare single cases that are not following the usual protocol. Analyzing those cases, it is possible to create pre-programmed contingency plans for such cases in order to be prepared and properly trained for the management of out-of-the-protocol cases. The PALIA ILS tool not only allows one to achieve better knowledge about the behavior of the deployed process, but also, it allows ILS technicians to detect inconsistencies in the localization protocol. The ILS technicians can use these data to iteratively correct the system to increase its accuracy.

In our opinion, the use of a web-based approach nearer to the user and integrated in their workflow has a better impact on the usability and understandability of the final user than the use of generalist applications. The results of the questionnaire show a good acceptance of the system, as well as a good perception of the utility of the knowledge acquired from the system. The creation of specifically-designed tools to deploy the process mining techniques can be a good alternative to approach the usability and understandability challenges of non-experts for process mining [43].

In addition, using other filters over the corpus can provide other interesting views about the process in order to achieve specific knowledge of some details of the process. For example, it is possible to infer the process flow of a single surgeon in order to compare it with others and to evaluate their compliance with the protocol or to analyze the process flow of patients with a specific illness. In this way, this paradigm can be very useful to achieve more and better findings for an increase the quality of service in healthcare centers.

In this work, we have focused on the use of process mining techniques for the analysis of the medical protocols. However, the use of ILS samples for feeding process mining algorithms allows not only an unobtrusive way to collect data from the process, but also allows the use of the physical model of the building to perform better conformance analysis of the events of the corpus. Using this physical model, it will be possible to use other process mining techniques, like escaping arcs [46], trace alignment [47] or token games [48], to have not only a better knowledge of the process, but also a better idea of the precision of the system. Currently, we are working with this approach in order to support the deployment of precise ILS systems.

In order to apply the results achieved in this study to any another context, it is necessary to deal with the spaghetti effect limitation. The spaghetti effect is a well-known effect that decrease the understandability of flows in very complex problems. According to our experiments [42], it is necessary to have adequate algorithms, workflow representation languages and visualization rendering for the problem to solve. This requires continued contact with health professionals in order to create better and more useful tools for supporting them in their daily practice.
